# Sabiá Virus–Like Mammarenavirus in Patient with Fatal Hemorrhagic Fever, Brazil, 2020

**DOI:** 10.3201/eid2606.200099

**Published:** 2020-06

**Authors:** Fernanda de Mello Malta, Deyvid Amgarten, Ana Catharina de Seixas Santos Nastri, Yeh-Li Ho, Luciana Vilas Boas Casadio, Marcela Basqueira, Gloria Selegatto, Murilo Castro Cervato, Amaro Nunes Duarte-Neto, Hermes Ryoiti Higashino, Felipe Arthur Faustino Medeiros, José Luiz Pinto Lima Gendler, Anna S. Levin, João Renato Rebello Pinho

**Affiliations:** Hospital Israelita Albert Einstein, São Paulo, Brazil (F.M. Malta, D. Amgarten, M. Basqueira, M.C. Cervato, J.R.R. Pinho);; Universidade de São Paulo, São Paulo (D. Amgarten, A.C. de Seixas Santos Nastri, Y.-L. Ho, L.V.R. Casadio, G. Selegatto, A.N. Duarte-Neto, H.R. Higashino, F.A.F. Medeiros, J.L.P.L. Gendler, A.S. Levin, J.R.R. Pinho)

**Keywords:** Viruses, Sabiá virus, arenaviruses, New World arenaviruses, viral hemorrhagic fever, metagenomics, Brazil, next-generation sequencing, mammarenavirus

## Abstract

New World arenaviruses can cause chronic infection in rodents and hemorrhagic fever in humans. We identified a Sabiá virus–like mammarenavirus in a patient with fatal hemorrhagic fever from São Paulo, Brazil. The virus was detected through virome enrichment and metagenomic next-generation sequencing technology.

Viral infections have become an important public health issue in South America during the past 2 decades. Outbreaks of arboviral disease, such as dengue, chikungunya, Zika, and yellow fever, have increased concern about improving surveillance and diagnosis of viral infections in anticipation of the next threat (*1*–*4*). South American arenaviruses belong to the New World serogroups, cause chronic infection in rodents, and have been associated with neurologic symptoms and hemorrhagic fever in humans (*5*).

We report a fatal case of a new Sabiá virus (SABV)–like mammarenavirus infection in São Paulo, Brazil, in a previously healthy 52-year-old man. On December 30, 2019, he sought care at a basic health facility for odynophagia, epigastric pain radiating to the chest, nausea, vertigo, xerostomy, and myalgia. During the next few days, his signs and symptoms worsened to intense myalgia, fever, drowsiness, and hypotension, so he was referred to the Hospital das Clinicas, Faculdade de Medicina da Universidade de São Paulo (São Paulo, Brazil), on January 6, 2020. At admission, he had bilateral conjunctivitis, diffuse skin rash, cervical lymphadenopathy, and altered mental status.

Blood samples were sent to the Laboratorio de Tecnicas Especiais (LATE), Hospital Israelita Albert Einstein (São Paulo, Brazil). Results of serologic and molecular tests were negative for yellow fever, dengue, Zika, and chikungunya viruses; viral hepatitis; enteroviruses; and herpesviruses. Further testing was performed with a newly implemented routine test for RNA viruses, a virome enrichment technique, and metagenomic next-generation sequencing (NGS) technology. In brief, total RNA was extracted from plasma, and human rRNA was depleted. Reverse transcription of RNA was performed with a 2-step cDNA random synthesis, followed by PCR amplification, adapted from Greninger et al. (*6*). We then generated DNA libraries using Illumina Nextera XT (Illumina, https://www.illumina.com) and sequenced them using Illumina NextSeq 550. We used the data generated as input for an in-house bioinformatics pipeline, performed as follows: raw data quality control, human decontamination, first round of pathogen identification, reads assembly, second round of identification with contigs, and finding confirmation through mapping and calculating quality metrics.

The patient’s condition deteriorated, and he died on January 11. Main autopsy findings were cerebral edema, hepatomegaly with steatotic aspect, and pulmonary hemorrhage. Microscopy showed panlobular hepatitis, with steatotic and apoptotic hepatocytes, hyperplasic Kupffer cells, and mild inflammatory reaction in the liver. Ischemic coagulative necrosis around central vein was also noted. Other findings included bronchopneumonia, acute tubular necrosis, and hemophagocytosis in the bone marrow. Detailed histologic studies are under way.

The report of the virome test from the patient’s plasma sample identified the presence of a mammarenavirus, SP2019-01. Further bioinformatics analysis enabled recovery of 2 complete fragments of the viral genome corresponding to the small (S [3,308 bp]) and large (L [7,056 bp]) segments of an arenavirus; NGS average coverage was 117× for the S and 1,146× for the L segment. The S segment had 87% identity and the L segment had 89% identity in a full nucleotide alignment with SABV strain SPH114202 (Figure, panel A). We submitted sequences to GenBank (accession nos. MN956773 [L segment] and MN956774 [S segment]). A maximum-likelihood tree of the species within the Arenaviridae family shows strain SP2019-01 as a SABV-like mammarenavirus based on the alignment of arenavirus complete nucleotide sequences of the L segment (Figure, panel B).

**Figure:**
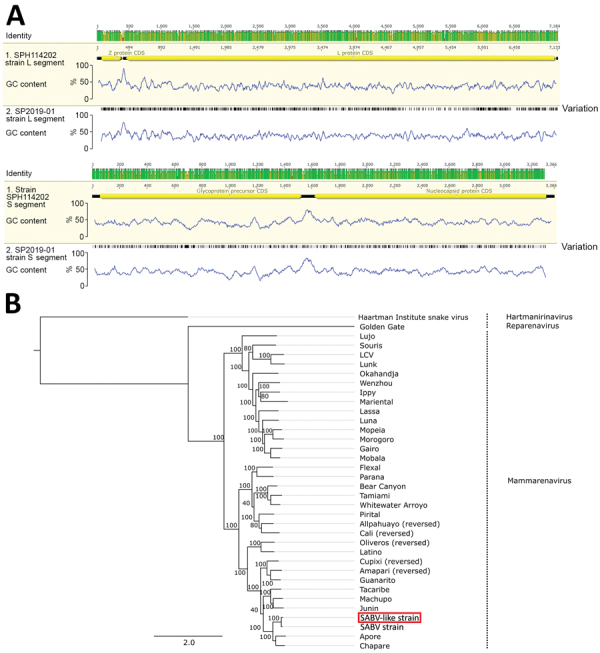
Genomic and phylogenetic analysis of SABV-like mammarenavirus (SP2019-01) from a patient with fatal hemorrhagic fever, Brazil, 2020. A) Genome plots comparing strain SP2019-01 with SABV strain SPH114202, showing identity throughout the genome and variant sites (black lines). B) Maximum-likelihood tree of SP2019-01 (red box) based on the alignment of arenavirus sequences. Tree was rooted in the Haartman Institute virus isolate sequence, and bootstrap values are shown next to the branches. CDS, coding sequence; GC, content of guanosine and cytosine; L, large; S, small; SABV, Sabiá virus. Scale bar indicates nucleotide substitutions per site.

SABV is a New World arenavirus that was previously isolated in São Paulo in 1994 in a patient with fatal hemorrhagic fever (*7*). Four additional New World arenavirus are known to be associated with human disease in South America: Junin, Machupo, Guanarito, and Chapare (*8*). Chapare virus is closely related to SABV and has been recently implicated in an outbreak of 5 cases (3 fatal) of hemorrhagic fever in Bolivia (*8*,*9*).

Little is known about the natural history of SABV infection. The wild reservoir species for this virus is still unknown but is probably a rodent (*10*). Three additional SABV cases in humans have been reported since 1994 including 2 in laboratory workers handling SABV samples, raising concern about the potential for aerosol transmission (*10*).

After the virome test report from this investigation was issued, the laboratory team took all measures to remove biological samples from laboratory routine testing and store them at −80°C. Persons who had any contact with the patient were informed and monitored and did not develop symptoms. Brazil health and surveillance authorities, as well as the US Centers for Disease Control and Prevention, were also informed. Our finding of an SABV-like arenavirus strain causing a fatal human infection in a region with a recent outbreak of yellow fever show the potential role of virome enrichment technique and metagenomic NGS for the etiologic diagnosis and identification of new pathogens in patients with hemorrhagic fever.

## References

[1] Morens DM, Fauci AS. Emerging infectious diseases: threats to human health and global stability. PLoS Pathog. 2013;9:e1003467. 10.1371/journal.ppat.1003467 23853589 PMC3701702

[2] Grubaugh ND, Ladner JT, Lemey P, Pybus OG, Rambaut A, Holmes EC, et al. Tracking virus outbreaks in the twenty-first century. Nat Microbiol. 2019;4:10–9. 10.1038/s41564-018-0296-2 30546099 PMC6345516

[3] Deng X, Achari A, Federman S, Yu G, Somasekar S, Bártolo I, et al. Metagenomic sequencing with spiked primer enrichment for viral diagnostics and genomic surveillance. [Erratum in: Nat Microbiol. 2020;5:525.]. Nat Microbiol. 2020;5:443–54. 10.1038/s41564-019-0637-9 31932713 PMC7047537

[4] Chong HY, Leow CY, Abdul Majeed AB, Leow CH. Flavivirus infection-A review of immunopathogenesis, immunological response, and immunodiagnosis. Virus Res. 2019;274:197770. 10.1016/j.virusres.2019.197770 31626874

[5] Brisse ME, Ly H. Hemorrhagic fever-causing arenaviruses: Lethal pathogens and potent immune suppressors. Front Immunol. 2019;10:372. 10.3389/fimmu.2019.00372 30918506 PMC6424867

[6] Greninger AL, Naccache SN, Federman S, Yu G, Mbala P, Bres V, et al. Rapid metagenomic identification of viral pathogens in clinical samples by real-time nanopore sequencing analysis. Genome Med. 2015;7:99. 10.1186/s13073-015-0220-9 26416663 PMC4587849

[7] Lisieux T, Coimbra M, Nassar ES, Burattini MN, de Souza LT, Ferreira I, et al. New arenavirus isolated in Brazil. Lancet. 1994;343:391–2. 10.1016/S0140-6736(94)91226-2 7905555 PMC3313646

[8] Delgado S, Erickson BR, Agudo R, Blair PJ, Vallejo E, Albariño CG, et al. Chapare virus, a newly discovered arenavirus isolated from a fatal hemorrhagic fever case in Bolivia. PLoS Pathog. 2008;4:e1000047. 10.1371/journal.ppat.1000047 18421377 PMC2277458

[9] Escalera-Antezana JP, Rodriguez-Villena OJ, Arancibia-Alba AW, Alvarado-Arnez LE, Bonilla-Aldana DK, Rodríguez-Morales AJ. Clinical features of fatal cases of Chapare virus hemorrhagic fever originating from rural La Paz, Bolivia, 2019: A cluster analysis. Travel Med Infect Dis. 2020;101589; Epub ahead of print. 10.1016/j.tmaid.2020.101589 32061859

[10] Ellwanger JH, Chies JAB. Keeping track of hidden dangers - The short history of the Sabiá virus. Rev Soc Bras Med Trop. 2017;50:3–8. 10.1590/0037-8682-0330-2016 28327796

